# Endoscopy and magnetic resonance imaging-based prediction of ypT stage in patients with rectal cancer who received chemoradiotherapy: Results from a prospective study of 110 patients

**DOI:** 10.1097/MD.0000000000016614

**Published:** 2019-08-30

**Authors:** Min Soo Cho, HonSoul Kim, Yoon Dae Han, Hyuk Hur, Byung Soh Min, Seung Hyuk Baik, Jae Hee Cheon, Joon Seok Lim, Kang Young Lee, Nam Kyu Kim

**Affiliations:** a Division of Colon and Rectal Surgery, Yonsei University College of Medicine; b Department of Radiology, Samsung Medical Center, Sungkyunkwan University School of Medicine; c Department of Internal Medicine and Institute of Gastroenterology; d Department of Radiology, Yonsei University College of Medicine, Seoul, Korea.

**Keywords:** chemoradiotherapy, endoscopy, magnetic resonance tumor regression grade, rectal cancer, tumor response, ypT stage

## Abstract

Accurate tumor response determination remains inconclusive after preoperative chemoradiation therapy (CRT) for rectal cancer. This study aimed to investigate whether clinical assessment, such as endoscopy and magnetic resonance imaging (MRI), can accurately predict ypT stage and select candidates for pelvic organ-preserving surgery in rectal cancer after preoperative CRT. A total of 110 patients who underwent preoperative CRT followed by curative resection for rectal cancer were prospectively enrolled. Magnetic resonance tumor regression grade (mrTRG) using T2-MRI, endoscopic evaluation, and combination modality (combination of endoscopy and mrTRG) were used to analyze tumor response after preoperative CRT. Endoscopic findings were categorized as 3 grades and the mrTRG was assessed into 5 grades. Twenty-nine patients (26.4%) had achieved pathologic complete response. When predicting ypT0, endoscopy showed significantly higher area under the curve (AUC 0.818) than did mrTRG (AUC 0.568) and combination modality (AUC 0.768) in differentiating good response from poor response (*P* < .001). Both endoscopy and combination modality showed significantly higher diagnostic performance in sensitivity (79.31%), positive predictive value (PPV 67.65%), negative predictive value (NPV 92.11%), and accuracy (84.55%) than those of MR tumor response (sensitivity 37.93%, PPV 36.67%, NPV 77.50%, and accuracy 66.36%) for the prediction of ypT0 (*P* < .001). Combination modality showed significantly higher diagnostic performance in sensitivity (56.92%), NPV (56.92%), and accuracy (67.27%) compared with those of mrTRG. Neither endoscopy, nor mrTRG, nor the combination modality had adequate diagnostic performances to be clinically acceptable in selecting candidates for nonoperative treatment strategies. However, endoscopy may be incorporated in clinical restaging strategy in planning the extent of surgical resection in patients with rectal cancer.

## 1 Introduction

With the progression of multidisciplinary approaches for the treatment of locally advanced rectal cancer (LARC), chemoradiation therapy (CRT) followed by total mesorectal excision (TME) has been widely adopted as a standard treatment for LARC.^[1]^ This treatment strategy provides a better outcome regarding sphincter preservation rates, the probability of curative resection, and reducing local recurrence rates. Undoubtedly, the primary endpoint of preoperative CRT is the achievement of pathologic complete response (pCR) prior to surgery. pCR, which is obtained in 8.0% to 24.0% of patients receiving preoperative CRT, is strongly associated with good long-term outcomes, and has been suggested as a prognostic indicator.^[2–6]^ In contrast, the majority of patients who received preoperative CRT showed significant residual disease, and these patients showed significantly poorer outcomes compared with patients with good tumor responses.^[3]^ Although pathologic response information can serve as a prognostic indicator, it may not be available before surgical removal of the tumor. Under these circumstances, preoperative prediction of pathologic response to preoperative CRT could enable development of personalized treatment protocols, reducing unnecessary exposure of patients to extensive surgery, and subsequently improving quality of life.

For these reasons, several studies using preoperative clinical or radiologic methods have focused on identifying reliable parameters to predict pathologic tumor response in patients with rectal cancer receiving preoperative CRT. The use of high-quality T2-weighted images for the evaluation of tumor regression grade (mrTRG), which is closely resembles the Mandard TRG grade, has been shown to be a reliable method for assessing tumor response prior to surgery.^[7,8]^ Other recent studies demonstrated that endoscopy can be easily performed and is more effective in assessing the intraluminal tumor remnant.^[9,10]^ Furthermore, these reports had led to a growing interest in sphincter-preserving surgery or a “wait-and-see” policy to avoid postoperative morbidities in a few studies.^[2,5,11]^

However, to date, accurate tumor response determination remains inconclusive, because radiation-induced fibrotic changes or inflammatory reactions in the rectum after CRT make it difficult to evaluate the tumor response accurately.^[12–14]^ Furthermore, the concordance between the clinically complete response (cCR) and pCR remains unclear.^[15–18]^

Over 3 years, we conducted a prospective evaluation of the correlation between clinical tumor response and pathologic tumor response in patients who underwent preoperative CRT followed by TME for LARC. The aim of this study was to investigate whether endoscopy, mrTRG, and combination modality can predict ypT status, and whether those findings can facilitate selection of potential candidates for pelvic organ-preserving surgery after preoperative CRT.

## 2 Patients and methods

### 2.1 Patient selection

**Figure 1:**
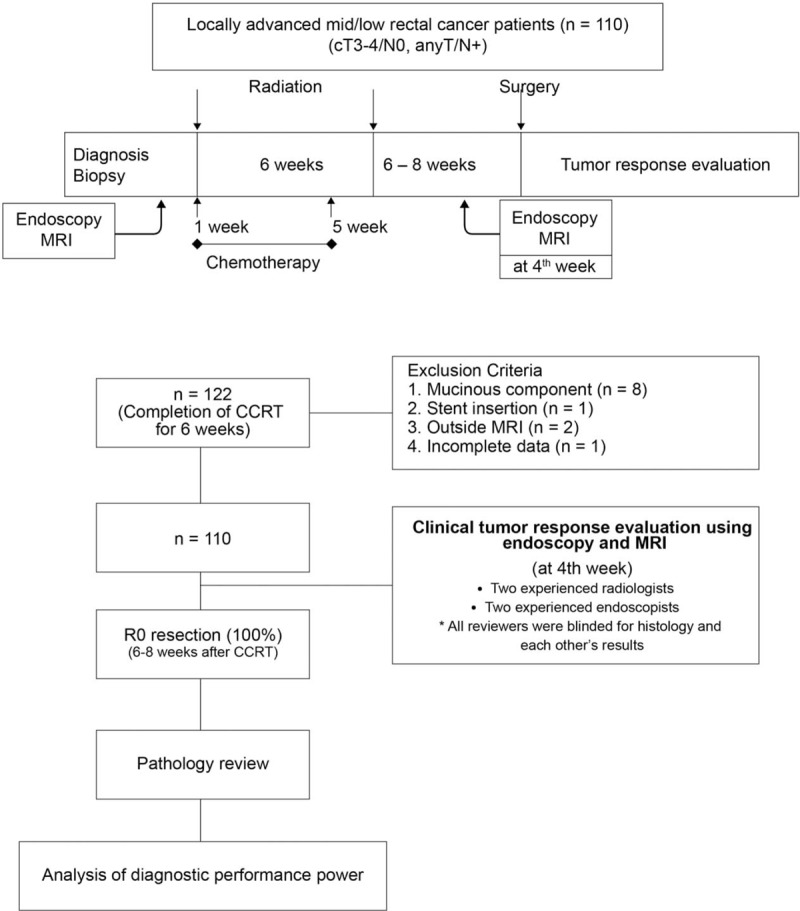
Flowchart of patient selection and study protocol. MRI = magnetic resonance imaging.

This prospective study was approved by the Institutional Review Board of Severance Hospital. Patients were enrolled between September 2011 and December 2013. Inclusion criteria were as follows: age >20 years; ECOG performance status ≥2; diagnosis of rectal adenocarcinoma via endoscopic biopsy, stage T3 or T4, or with clinically enlarged regional lymph nodes identified by CT or rectal magnetic resonance imaging (MRI) regardless of T stage; and having completed preoperative CRT followed by TME with curative intent. Exclusion criteria were as follows: stage IV disease; familial adenomatous polyposis or hereditary nonpolyposis colorectal cancer; benign disease; tumor located more than 10.0 cm from the anal verge; previous or concurrent malignant disease; and patient refusal. Of 122 consecutive patients originally recruited, 12 patients were initially excluded for the analysis: 8 with a mucinous component by preoperative rectal MRI, 2 with incomplete MRI protocols, 1 with stent insertion for an obstructive lesion, and 1 with incomplete data. The remaining 110 patients who fulfilled the inclusion criteria were finally included (Fig. 1).

### 2.2 Endoscopic assessment of tumor response before and after preoperative CRT

**Figure 2:**
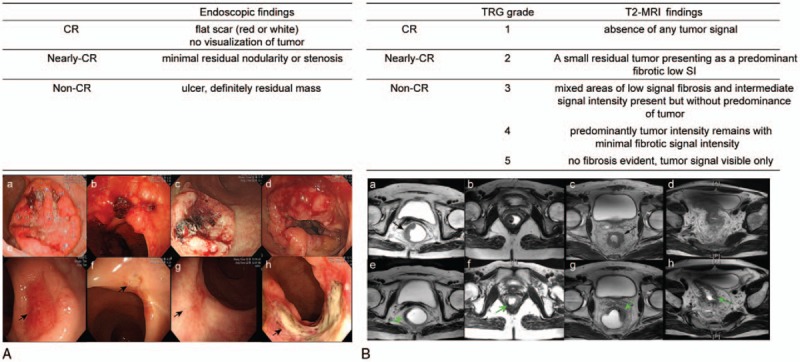
(A) Tumor on endoscopy before and after preoperative chemoradiation therapy (pre-CRT). (a–d) Primary tumor before pre-CRT. (e, f) Clinically complete response of primary tumor after completion of pre-CRT; a typical sign of clinical complete response (black arrows indicate a red scar and a white scar). (g) Minimal residual nodularity and (h) residual tumor with large necrotic area after pre-CRT (a typical sign of poor tumor response). (B) Magnetic resonance imaging (MRI) scans from patients before and after pre-CRT. (a–d) Primary tumor before pre-CRT. (e) Tumor regression grade (TRG) 1; the green arrow indicates radiologic complete response with a hypointense rectal wall. (f) TRG 2; the green arrow indicates a slightly thickened hypointense rectal wall. (g) TRG 3; the green arrow indicates a visible intermediate signal. (h) TRG 4; the green arrow indicates a small area of fibrosis.

All endoscopic procedures were performed by gastrointestinal endoscopists with more than 5 years’ experience. A pre-CRT endoscopy, accompanied by tissue biopsy, was performed before initiating preoperative CRT. Four weeks after the completion of preoperative CRT, endoscopic tumor response was evaluated prior to surgery (Fig. 1). All reviewers were blinded for histology and each other's results. Under same categorical criteria, all reviewers conducted reassessment of tumor response considering the tumor size, morphology, and involved intraluminal circumference before surgery. Endoscopic tumor response was categorized as follows: cCR, no visualization of tumor, white or red scar; nearly-cCR, minimal residual nodularity or stenosis; non-cCR, any ulcer with a necrotic bed regardless size, a definite residual mass, or nodularity (Fig. 2A).

### 2.3 MRI assessment of tumor response before and after preoperative CRT

All magnetic resonance images were obtained using a 3-T scanner (Magnetom Tim Trio, Siemens Medical Solutions, Erlangen, Germany) with a 6-element body phased-array coil on the anterior side of the patient and another 6 elements on the spine coil on the posterior side. For optimal rectal distension, 80 to 100 mL of sonography transmission gel was administered endorectally using an enema syringe. Two experienced gastrointestinal radiologists evaluated MR tumor response after preoperative CRT, and determined TRG before surgery. The mrTRG was assessed into 5 grades. mrTRG1: the absence of any residual tumor lesion; mrTRG2: a small residual tumor presenting as a predominant fibrotic low SI; mrTRG3: all lesions showing partial decrease in size upon comparison of pre-/post-CRT MRIs, but not meeting the criteria of mrTRG 1 or 2; and mrTRG 4 and 5 were each graded if the tumor did not decrease in size, or progressed, respectively.^[19]^ MRI findings were finally categorized according to mrTRG: cCR, TRG1; nearly-cCR, TRG2; non-cCR, TRG3-5 (Fig. 2B). The median time to restaging was 4.1 weeks (interquartile range 3.9–4.3 weeks).

### 2.4 Preoperative chemoradiation therapy protocol and surgical treatments

We used a standard long-course regimen of 5-fluorouracil (5-FU)-based chemotherapy and a total dose of 50.4 Gy of external beam radiation. Preoperative chemotherapy of 425 mg/m^2^ 5-FU per day and 20 mg/m^2^ leucovorin per day during weeks 1 and 5 of radiotherapy was administered intravenously. After a median interval of 7.8 weeks (interquartile range: 6.4–8.4 weeks) from completion of CRT, all patients underwent radical cancer resection, based on the principles of TME.

### 2.5 Histopathologic assessment

The surgical specimens were prepared and dissected according to the protocol described by Nagtegaal et al.^[20]^ The resection surface of the mesorectum and the specimen was fixed in formalin for a minimum of 48 hours. Dissection consisted of serial 5- to 10-mm slicing of the whole tumor and the surrounding mesorectum in the transverse plane. Specimens were embedded in paraffin for histologic examination and stained with hematoxylin and eosin. For all tumors, the shortest distance from the outermost part of the tumor to the circumferential resection margin (CRM) was measured histologically. All tumors were staged according to the 7th American Joint Committee on Cancer (AJCC) TNM classification. At pathologic examination, the response was considered to be major for tumors classified ypT1 or ypT2, and complete (ypT0) when no viable tumor was present.

### 2.6 Data and statistical analysis

We calculated a sample size of 110 patients in this study, based on a rate of ypT0 of 20% observed in previous studies. The power was set at 80% and the 2-sided significance level set at 0.05, and the sample size was inflated by 10% to account for an ineligibility rate of 10%. Pathologic tumor response to CRT was divided into good and poor response groups using 3 different criteria as follows: ypT0 vs ypT1-4; ypT0-1 vs ypT2-4; or ypT0-2 vs ypT3-4. In the analysis of diagnostic performance of endoscopic and MR tumor response for predicting the good response group, post-CRT findings were scored on a 3-point scale: 3 for cCR, 2 for nearly-cCR, and 1 for non-cCR. For both endoscopic and MR tumor response, positive test results were defined as a score of 2 or higher and negative results were defined as a score of 1. For the combination of endoscopic and MR tumor response (combination modality), positive test results were defined as the sum of these 2 methods scores equaling 3 or higher and negative results were defined as the sum of the scores equaling 2. The sensitivities, specificities, positive predictive values (PPVs), negative predictive values (NPVs), and accuracy of endoscopic tumor response, MR tumor response, and the combination modality were calculated for differentiating the good response from the poor response groups, when applying each definition for the good response group (ypT0, ypT0-1, or ypT0-2). The area under the curves (AUCs) were also calculated from construction of the receiver operating characteristic (ROC) curve.^[21]^ Pairwise comparison of AUCs of endoscopic tumor response, MR tumor response, and the combination modality were performed (endoscopy vs mrTRG, endoscopy vs combination modality, mrTRG vs combination modality). Generalized estimating equation (GEE) model was used to obtain statistically unbiased estimates. SPSS software version 20.0 for Windows (SPSS Corp, Chicago, IL) was used for analyses. Quantitative data were expressed as mean ± standard deviation and qualitative data as frequency and percent. All *P*-values were 2-sided, and *P* < .05 was considered statistically significant.

## 3 Results

### 3.1 Patient characteristics

The patient characteristics and operative data are shown in Table 1. The mean age of all 110 patients was 59.3 ± 11.9 years. The mean distance between the tumor and the dentate line was 5.6 ± 2.8 cm. Five of the 110 patients had a very low rectal tumor, which involved the dentate line and was classified as cT2N+ on preoperative evaluation. These patients received preoperative CRT to increase the possibility of a sphincter-preserving surgery. The mean time interval to surgery was 5.6 ± 2.8 weeks between completion of CRT and primary surgery. A sphincter-preserving surgery was performed in 102 (92.7%) patients. The remaining 8 patients underwent abdominoperineal resection for a very low tethered rectal tumor, which did not respond sufficiently to preoperative CRT. Ninety-nine (90%) patients underwent minimally invasive surgery (MIS), including laparoscopic and robotic surgery. There was no conversion to open surgery in patients who underwent MIS.

**Table 1:**
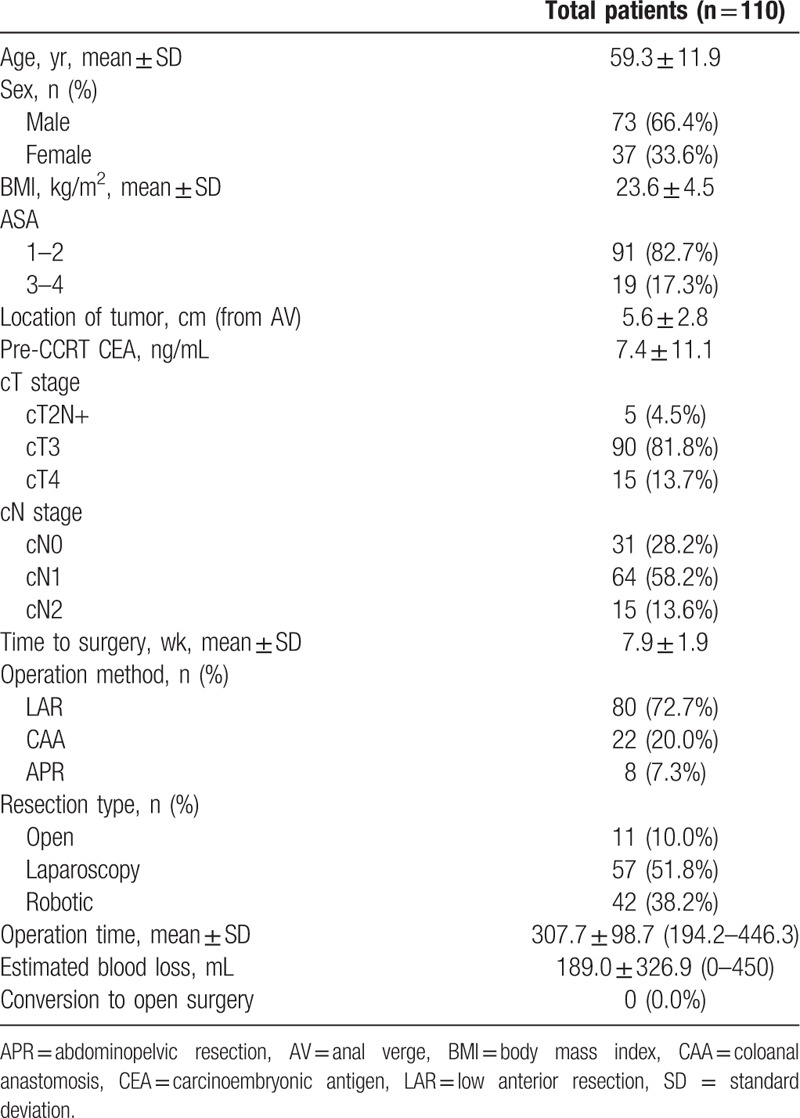
Patients characteristics and operative data.

### 3.2 Postoperative pathologic outcomes

At pathologic examination, ypT0, ypT0-1, and ypT0-T2 were observed in 30 (27.3%), 39 (35.5%), and 65 (59.1%) cases, respectively. Lymph node metastasis was observed in 34 cases (30.9%). The proportion of patients with a positive CRM was 2.7% in all patients. Of patients who underwent preoperative CRT followed by TME, 29 patients (26.4%) achieved a pCR (Table 2). T downstage was defined as the downstage of the tumor from cT2 to ypT0-1 or cT3 to ypT0-2 or from cT4 to ypT0-3. In all, 68 patients (61.8%, 68/110) were found to have a T downstage after preoperative CRT, and 57 patients (57.1%, 60/105) had less than ypT2 stage in patients with cT3-4 stage.

**Table 2:**
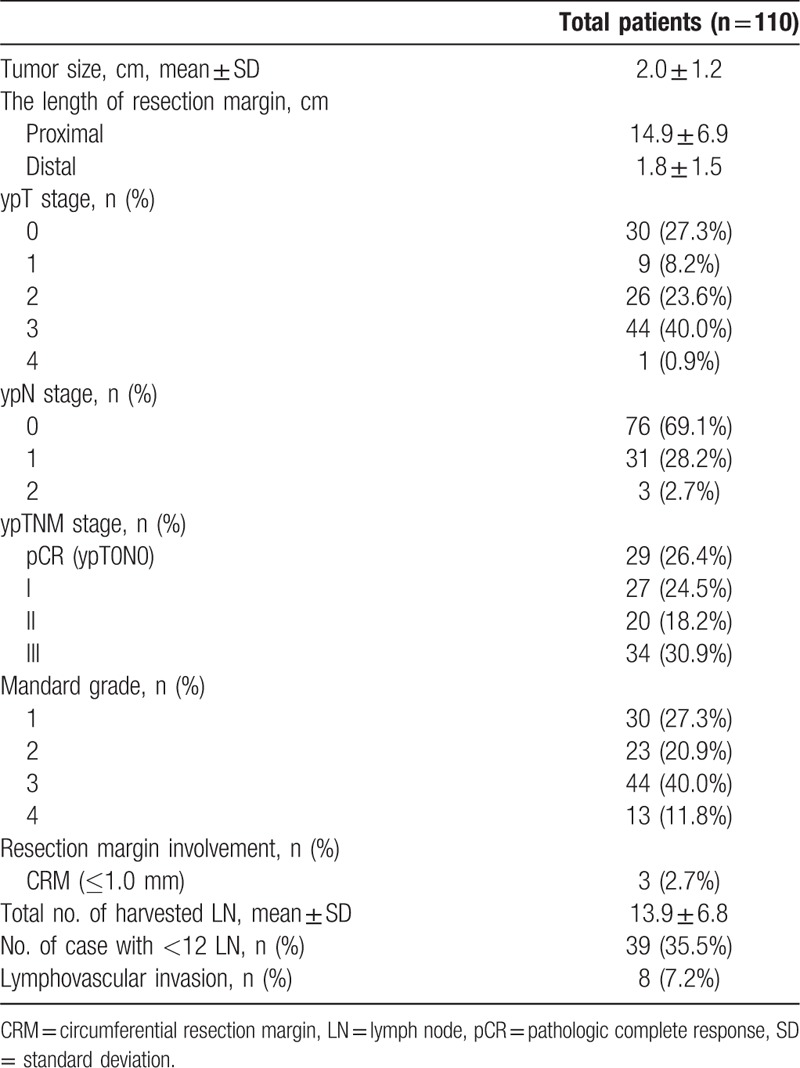
Postoperative pathologic outcomes.

### 3.3 Endoscopy, mrTRG, and combination modality for predicting the good-response group using ROC curves

**Figure 3:**
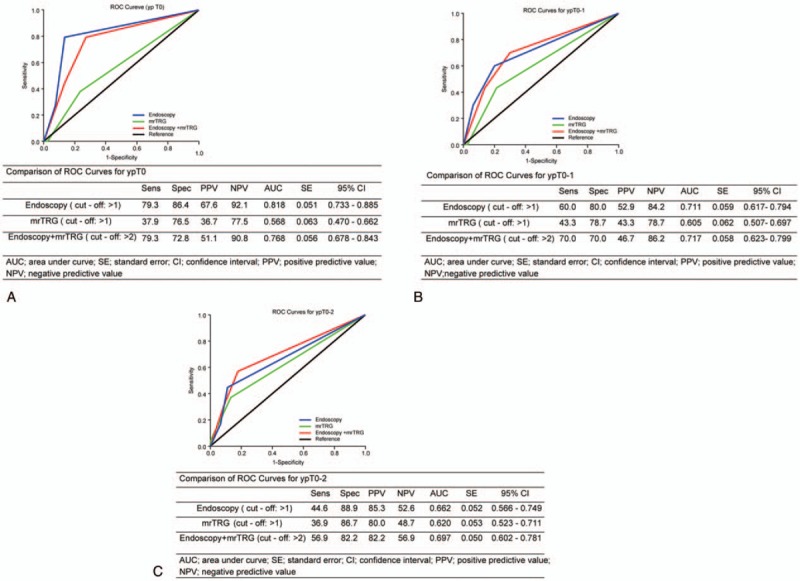
(A) Comparison of receiver operating characteristic (ROC) curves according to endoscopy, magnetic resonance tumor regression grade (mrTRG), and the combination modality (endoscopy added to mrTRG) for predicting ypT0. (B) Comparison of ROC curves predicting ypT0-1. (C) Comparison of ROC curves predicting ypT0-2.

When the good response group was defined as ypT0 (Fig. 3A), the values of AUC showed significant differences between the 3 modalities (*P* < .001). Endoscopy showed significantly higher AUC (0.818) than did mrTRG and combination modality in differentiating good response from poor response (*P* < .001). Among the 3 modalities, endoscopy showed the highest value and mrTRG showed the lowest value. When the good response group was defined as ypT0-1 (Fig. 3B), the 3 modalities showed no significant differences in differentiating good tumor response from poor response (*P* = .117). However, the AUC (0.717) of the combination modality was significantly higher than that of mrTRG (*P* = .011). When the good response group was defined as ypT0-2 (Fig. 3C), the 3 modalities showed significant differences in differentiating good tumor response from poor response (*P* = .011). The AUC (0.697) of the combination modality was the highest among the methods, and it was significantly higher than that of mrTRG (*P* = .026).

### 3.4 Diagnostic performance of each modality in the evaluation of tumor response to chemoradiation using GEE

**Figure 4:**
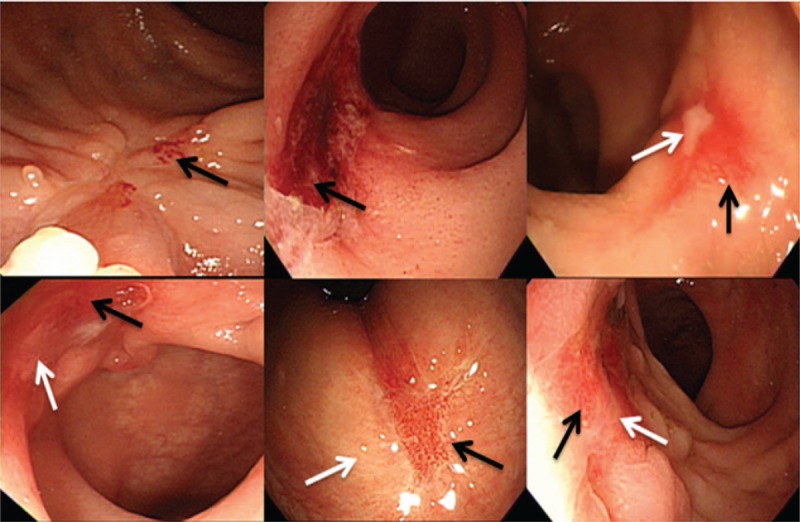
Response assessment with endoscopy; red scar with telangiectasia (black arrow) and white scar (white arrow) shows typical sign of clinical complete response after chemoradiation therapy.

Tables 3 to 5 show the diagnostic performance of between the 3 modalities and ypT stages of 110 tumors. Among a total of 110 patients, 34 patients showed good tumor response by both the endoscopy and combination modality (Fig. 4). Of these 34 patients, 23 were confirmed as true absence of tumor by histopathologic analysis, which meant that the PPV of endoscopy for ypT0 was 67.65% (95% confidence interval [CI]: 51.92–83.37) by using the GEE. On the contrary, 11 of 30 patients with good tumor response by MR tumor response were confirmed as ypT0 by histopathologic analysis (PPV 36.67%, 95% CI: 19.42–53.91). Both endoscopy and combination modality showed the highest values in sensitivity (79.31%), PPV (67.65%), NPV (92.11%), and accuracy (84.55%) and theses values showed significantly higher diagnostic performances than those of mrTRG for the prediction of ypT0 (Table 3). When evaluating ypT0-1 by using 3 modalities, there was no significant difference at distinguishing ypT0–1 from ypT2–4 (PPV of endoscopy: 52.94% (95% CI: 26.16–69.72), PPV of mrTRG: 43.33% (95% CI: 25.60–61.07), PPV of combination modality: 46.67% (95% CI: 32.09–61.24), respectively, *P* = .330). However, combination modality showed significantly higher sensitivity than that of mrTRG (70.00% vs 43.33%, *P* = .001) (Table 4). When comparing 3 modalities for the prediction of ypT0-2, all modalities showed relatively high diagnostic performance in differentiating ypT0-2 from ypT3-4 (PPV of endoscopy: 85.29% [95% CI: 73.39–97.20], PPV of mrTRG: 80.00% [95% CI: 65.69–94.31], PPV of combination modality: 82.22% [95% CI: 71.05–93.39], respectively, *P* = .688). In addition, sensitivity, NPV, and accuracy showed significantly higher values in combination modality than those of mrTRG (sensitivity: 56.92% vs 36.92%, *P* ≤ .001, NPV: 56.92% vs 48.75%, *P* = .003, accuracy: 67.27% vs 57.27%, *P* = .003, respectively) (Table 5).

**Table 3:**
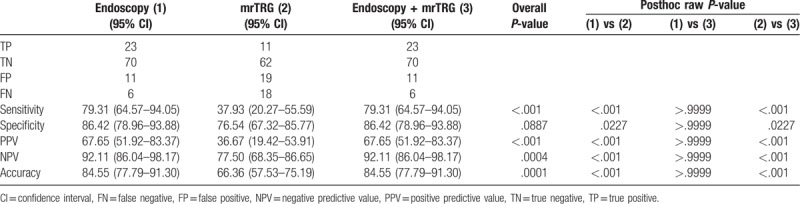
Diagnostic performance power predicting ypT0.

**Table 4:**
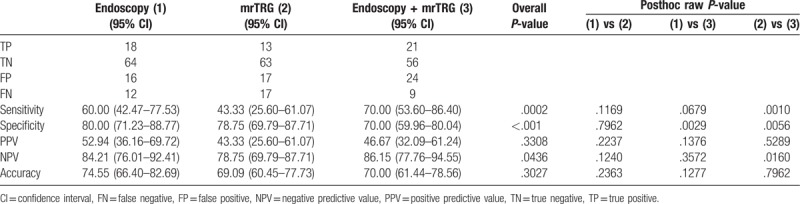
Diagnostic performance power predicting ypT0-1.

**Table 5:**
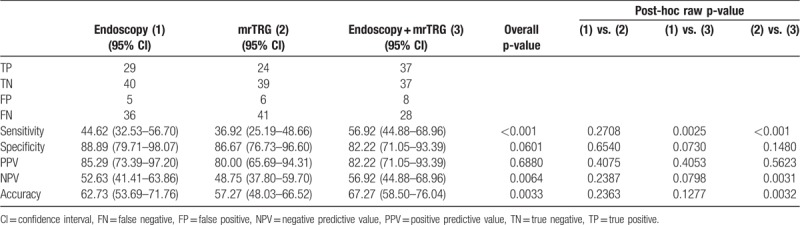
Diagnostic performance power predicting ypT0-2.

## 4 Discussion

The point of this study is to investigate the possibility of changing the planned surgery and the chance of providing organ-preserving surgery based on prediction of ypT stage. However, diagnostic performance power, based on a review of post-CRT endoscopic and mrTRG, did not distinguish ypT0-2 from ypT3-4, accurately. Nevertheless, endoscopic evaluation itself can be potentially considered as a new restaging modality with MRI. In rectal cancer surgery, many surgeons have been faced with a difficult decision about whether to perform organ-preserving surgery or extensive surgery when restaging tumor response in middle and lower rectal cancer after CRT. Some authors argued for the so-called “wait-and-see” policy in patients having a clinical CR based on clinical or radiologic assessment,^[2,5,11]^ and another authors reported that when patients had cT1 or cT2 after CRT, sphincter-preserving surgery or local excision could be considered.^[22–24]^ Although various imaging modalities, including MRI, endoscopic rectal ultrasound, computed tomography, and positron emission tomography, have been used to evaluate tumor response following CRT, most studies had limited accuracy to predict tumor response after CRT.^[25,26]^ Although limited, this study showed that the PPVs of endoscopy and mrTRG were 67.6% and 36.7% for predicting ypT0, and 52.9% and 43.3% for predicting ypT0-1. In addition, the PPVs of combination modality were 67.6% and 46.6% for predicting ypT0 and ypT0-1, respectively. In our series, 29 (26.4%) patients had pCR (ypT0N0) after the completion of preoperative CRT. This finding is consistent with previous studies. As mentioned before, a few limited studies reported that a wait-and-see policy with strict selection criteria resulted in a promising long-term oncologic outcome, at least as good as that of patients with pCR after radical surgery. However, so far, the use of such nonoperative treatment strategies remains inconclusive, and they are not regarded as the standard treatment even in patients with a pCR on the basis of the current guidelines. In our study, when predicting ypT0, endoscopy showed significantly higher AUC (0.818) than those of mrTRG (AUC 0.568) and the combination modality (AUC 0.768). Although endoscopy had relatively high diagnostic performance, these results reflected that neither endoscopy, nor mrTRG, nor the combination modality had adequate positive or negative predictive value to be clinically acceptable in selecting candidates for nonoperative treatment strategies. These findings reflect that a substantial number of patients may be treated inappropriately when applying “wait and see” management for patients who were preoperatively determined to have a cCR. Recently, Kawai et al demonstrated that nonoperative management was associated with the high rate of local failure (41.7%) in patients who had clinical CR with endoscopic assessment.^[27]^ Accordingly, given our results, nonoperative management based on clinical assessment should be considered carefully for highly selected patients.

On the contrary, many clinicians still argue that local excision of the residual scar after preoperative CRT is a much safer approach than a wait-and-see policy, because it provides reassurance about the presence or absence of remnant tumor. Although it is undoubtedly true that it accurately provides more opportunity to assess the tumor remnant, it could be a challenge for the surgeon to identify the precise site of the initial tumor needed to achieve an adequate resection margin, because scarring changes of the primary tumor with unclear landmarks make it difficult to identify microscopic residual tumor. From a similar point of view, this study showed that none of 3 modalities had reliable PPVs to distinguish ypT0-1 from ypT3-4. Furthermore, a few studies reported that there was a still 5.0% to 10.0% incidence of positive lymph nodes in ypT0-1 patients,^[2,28–31]^ and there is no clear consensus about the standard criteria to determine lymph node positivity. Therefore, when considering local excision or nonoperative treatment, treatment strategy should be cautiously determined due to the risk of leaving positive regional lymph nodes.

The Response Evaluation Criteria in Solid Tumors guidelines recommend that MRI is currently the best available method to evaluate lesions selected for response assessment.^[32]^ Most previous studies suggested that MRI can be useful for predicting pCR in patients who achieved a major radiologic response on MRI than in those who did not.^[33–35]^ However, when restaging rectal cancer after CRT, the utility of MRI remains debatable in terms of its accuracy, due to both overestimation and underestimation. In contrast, endoscopy is not advised for objective tumor evaluation in this guideline. To date, a few limited studies suggested that endoscopy may be effective with direct inspection of tumor burden for evaluating pathologic T stage.^[9,10]^ In other words, disappearance of the tumor with healing of the mucosa, decrease in size, and complete normalization of the tumor bed may be considered the typical sign of cCR or good tumor response. Based on these typical findings, our results showed that overall diagnostic performances of endoscopy were significantly higher than those of mrTRG in predicting the good response group. However, endoscopy potentially has inherent limitations in differentiating residual tumor from good tumor response, because endoscopic tumor response only provide gross information on the luminal area and not on the deeper bowel layers. Furthermore, a recent study demonstrated that residual tumor cells in the surgical specimen after CRT are frequently located close to the invasive front of the primary tumor.^[36]^ Therefore, the risk of underestimating scattered residual tumor cells always exists.

On the contray, unlike our expectation, overall diagnostic performance of the combination modality was not superior to endoscopy for the prediction of good tumor response. The reason for nonsynergistic effects of the combination modality may be explained by that the difficulties of anatomic imaging to adequately distinguish fibrotic tissue or local inflammation from residual tumor, making it difficult to evaluate tumor response accurately.^[14]^ Accordingly, these inherent limitations may adversely affect the correlation between endoscopy and mrTRG. However, both endoscopy and combination modality had significantly higher PPVs for the prediction of ypT0 and ypT0-2 than those of MR tumor response. However, none of these methods did guarantee that a patient had good tumor response for definite candidates for organ-preserving surgery in current clinical practice. We think that future technical advances in endoscopy or mrTRG (e.g., pit pattern or magnified endoscopy, or a high Tesla magnetic field strength [7.0 T] system) may make it possible to preoperatively select candidates who can be treated by organ-preserving surgery, although these methods show limited diagnostic performance currently.

Our study had some limitations. First, lymph node evaluation was not considered in this study because our study focused on only pathologic T stage to identify candidates for organ-preserving surgery. Second, our timing of restaging after pre-CRT (median time 4.1 weeks) is relatively short compared with the majority of other studies, which varied from 6 to 10 weeks. Although some authors suggested a minimum of 6 to 8 weeks and a mean of 6.5 weeks after pre-CRT for restaging,^[2,37]^ currently there is no standardized guideline in the timing of assessment. Recently, data from a meta-analysis reported that a longer interval, with more than 6 to 8 weeks was found to have significant increase of pCR rate by 6.0% compared with a shorter interval with <6 to 8 weeks.^[38]^ In our study, all patients underwent radical surgery after a median interval of 7.8 weeks (interquartile range: 6.4–8.4 weeks) from completion of CRT to avoid confusing results due to worsening fibrosis. Finally, interobserver variability may have existed among both radiologists and endoscopists. Although all reviewers who participated in the tumor restaging were fully experienced, this may be a potential confounding factor when evaluating the accuracy of the restaging.

In conclusion, although endoscopic tumor response could not accurately predict ypT stage with adequate accuracy to be considered clinically acceptable, endoscopy showed a relatively high diagnostic performance in distinguishing good tumor responder than that of MRI. This prospective study suggests that endoscopy may be potentially incorporated in the clinical restaging strategy when evaluating tumor response after preoperative CRT.

## Author contributions

**Conceptualization:** Min Soo Cho.

**Investigation:** Min Soo Cho, HonSoul Kim.

**Methodology:** Min Soo Cho, HonSoul Kim.

**Resources:** Min Soo Cho, HonSoul Kim.

**Supervision:** Hyuk Hur, Byung Soh Min, Seung Hyuk Baik, Jae Hee Cheon, Joon Seok Lim, Kang Young Lee, Nam Kyu Kim.

**Validation:** Min Soo Cho, HonSoul Kim, Hyuk Hur.

**Writing – original draft:** Min Soo Cho.

**Writing – review & editing:** Min Soo Cho, HonSoul Kim, Hyuk Hur, Yoon Dae Han, Byung Soh Min, Seung Hyuk Baik, Jae Hee Cheon, Joon Seok Lim, Kang Young Lee, Nam Kyu Kim.
